# Chinese expert consensus on the multidisciplinary management of pneumonitis associated with immune checkpoint inhibitor

**DOI:** 10.1111/1759-7714.14693

**Published:** 2022-10-21

**Authors:** Wenxian Wang, Qian Wang, Chunwei Xu, Ziming Li, Zhengbo Song, Yongchang Zhang, Xiuyu Cai, Shirong Zhang, Bin Lian, Wen Li, Anwen Liu, Ping Zhan, Hongbing Liu, Tangfeng Lv, Liyun Miao, Lingfeng Min, Yu Chen, Jingping Yuan, Feng Wang, Zhansheng Jiang, Gen Lin, Xingxiang Pu, Chuangzhou Rao, Dongqing Lv, Zongyang Yu, Xiaoyan Li, Chuanhao Tang, Chengzhi Zhou, Congying Xie, Junping Zhang, Hui Guo, Qian Chu, Rui Meng, Jingxun Wu, Rui Zhang, Liping Wang, Youcai Zhu, Xiao Hu, Yanru Xie, Xinqing Lin, Jing Cai, Fen Lan, Huijing Feng, Lin Wang, Wang Yao, Xuefei Shi, Jianhui Huang, Huafei Chen, Yinbin Zhang, Pingli Sun, Bing Wan, Fei Pang, Zanmei Xu, Kai Wang, Yuanli Xia, Mingxiang Ye, Dong Wang, Qing Wei, Shuitu Feng, Jianya Zhou, Jiexia Zhang, Donglai Lv, Wenbin Gao, Jing Kang, Genhua Yu, Xianbin Liang, Chengtao Yu, Lin Shi, Nong Yang, Lin Wu, Zhuan Hong, Wei Hong, Meiyu Fang, Yiping Zhang, Yuanzhi Lu, Guansong Wang, Shenglin Ma, Lu Si, Wenfeng Fang, Yong Song

**Affiliations:** ^1^ Department of Chemotherapy Chinese Academy of Sciences University Cancer Hospital (Zhejiang Cancer Hospital) Hangzhou China; ^2^ Department of Respiratory Medicine Affiliated Hospital of Nanjing University of Chinese Medicine, Jiangsu Province Hospital of Chinese Medicine Nanjing China; ^3^ Institute of Cancer and Basic Medicine (ICBM) Chinese Academy of Sciences Hangzhou China; ^4^ Department of Respiratory Medicine Affiliated Jinling Hospital, Medical School of Nanjing University Nanjing China; ^5^ Department of Shanghai Lung Cancer Center Shanghai Chest Hospital, Shanghai Jiao Tong University Shanghai China; ^6^ Department of Medical Oncology, Lung Cancer and Gastrointestinal Unit, Hunan Cancer Hospital/The Affiliated Cancer Hospital of Xiangya School of Medicine Central South University Changsha China; ^7^ Department of VIP Inpatient Sun Yet‐Sen University Cancer Center, State Key Laboratory of Oncology in South China, Collaborative Innovation Center for Cancer Medicine Guangzhou China; ^8^ Translational Medicine Research Center, Key Laboratory of Clinical Cancer Pharmacology and Toxicology Research of Zhejiang Province Affiliated Hangzhou First People's Hospital, Cancer Center, Zhejiang University School of Medicine Hangzhou China; ^9^ Key Laboratory of Carcinogenesis and Translational Research (Ministry of Education/Beijing), Department of Melanoma and Sarcoma Peking University Cancer Hospital and Institute Beijing China; ^10^ Key Laboratory of Respiratory Disease of Zhejiang Province, Department of Respiratory and Critical Care Medicine Second Affiliated Hospital of Zhejiang University School of Medicine, Cancer Center, Zhejiang University Hangzhou China; ^11^ Department of Oncology Second Affiliated Hospital of Nanchang University Nanchang China; ^12^ Department of Respiratory Medicine Affiliated Drum Tower Hospital, Medical School of Nanjing University Nanjing China; ^13^ Department of Respiratory Medicine Clinical Medical School of Yangzhou University, Subei People's Hospital of Jiangsu Province Yangzhou China; ^14^ Department of Medical Oncology Fujian Medical University Cancer Hospital and Fujian Cancer Hospital Fuzhou China; ^15^ Department of Pathology Renmin Hospital of Wuhan University Wuhan China; ^16^ Department of Internal Medicine, Cancer Center of PLA, Qinhuai Medical Area Affiliated Jinling Hospital, Medical School of Nanjing University Nanjing China; ^17^ Derpartment of Integrative Oncology Tianjin Medical University Cancer Institute and Hospital Tianjin China; ^18^ Department of Medical Oncology, Lung Cancer, and Hunan Cancer Hospital/The Affiliated Cancer Hospital of Xiangya School of Medicine Central South University Changsha China; ^19^ Department of Radiotherapy and Chemotherapy, Hwamei Hospital University of Chinese Academy of Sciences Ningbo China; ^20^ Department of Pulmonary Medicine Taizhou Hospital of Wenzhou Medical University Taizhou China; ^21^ Department of Respiratory Medicine, The 900th Hospital of the Joint Logistics Team (The Former Fuzhou General Hospital) Fujian Medical University Fuzhou China; ^22^ Department of Oncology Beijing Tiantan Hospital, Capital Medical University Beijing China; ^23^ Department of Medical Oncology Peking University International Hospital Beijing China; ^24^ State Key Laboratory of Respiratory Disease, National Clinical Research Center for Respiratory Disease Guangzhou Institute of Respiratory Health, The First Affiliated Hospital of Guangzhou Medical University (The First Affiliated Hospital of Guangzhou Medical University) Guangzhou China; ^25^ Department of Radiation Oncology First Affiliated Hospital, Wenzhou Medical University Wenzhou China; ^26^ Department of Thoracic Oncology, Shanxi Academy of Medical Sciences Shanxi Bethune Hospital Taiyuan China; ^27^ Department of Medical Oncology The First Affiliated Hospital of Xi'an Jiaotong University Xi'an China; ^28^ Department of Oncology Tongji Hospital of Tongji Medical College, Huazhong University of Science and Technology Wuhan China; ^29^ Cancer Center, Union Hospital, Tongji Medical College Huazhong University of Science and Technology Wuhan China; ^30^ Department of Medical Oncology, The First Affiliated Hospital of Medicine Xiamen University Xiamen China; ^31^ Department of Medical Oncology Cancer Hospital of China Medical University Shenyang China; ^32^ Department of Oncology Baotou Cancer Hospital Baotou China; ^33^ Department of Thoracic Disease Diagnosis and Treatment Center Zhejiang Rongjun Hospital, The Third Affiliated Hospital of Jiaxing University Jiaxing China; ^34^ Zhejiang Key Laboratory of Radiation Oncology Cancer Hospital of the University of Chinese Academy of Sciences (Zhejiang Cancer Hospital) Hangzhou China; ^35^ Department of Oncology Lishui Municipal Central Hospital Lishui China; ^36^ Department of Pathology Shanxi Academy of Medical Sciences, Shanxi Bethune Hospital Taiyuan China; ^37^ Department of Interventional Oncology, The First Affiliated Hospital Sun Yat‐sen University Guangzhou China; ^38^ Department of Respiratory Medicine Huzhou Hospital, Zhejiang University School of Medicine Huzhou China; ^39^ Department of Oncology, The Second Affiliated Hospital of Medical College Xi'an Jiaotong University Xi'an China; ^40^ Department of Pathology The Second Hospital of Jilin University Changchun China; ^41^ Department of Respiratory Medicine The Affiliated Jiangning Hospital of Nanjing Medical University Nanjing China; ^42^ Department of Medical Shanghai OrigiMed Co. Ltd Shanghai China; ^43^ Department of Medical Affairs AstraZeneca China Shanghai China; ^44^ Department of Oncology Xiamen Haicang Hospital Xiamen China; ^45^ Department of Respiratory Disease, Thoracic Disease Center, The First Affiliated Hospital, School of Medicine Zhejiang University Hangzhou China; ^46^ Department of Clinical Oncology The 901 Hospital of Joint Logistics Support Force of People Liberation Army Hefei China; ^47^ Department of Oncology The Third Affiliated Hospital of Shenzhen University Shenzhen China; ^48^ Guangdong Lung Cancer Institute, Guangdong Provincial Laboratory of Translational Medicine in Lung Cancer Guangdong Provincial People's Hospital, Guangdong Academy of Medical Sciences, School of Medicine Guangzhou China; ^49^ Department of Radiation Oncology Zhebei Mingzhou Hospital Huzhou China; ^50^ Department of Oncology The Third People's Hospital of Zhengzhou Zhengzhou China; ^51^ Collaborative Innovation Center of Jiangsu Province of Cancer Prevention and Treatment of Chinese Medicine Nanjing China; ^52^ Department of Respiratory Medicine Zhongshan Hospital, Fudan University Shanghai China; ^53^ Department of Medical Oncology Jiangsu Cancer Hospital, Nanjing Medical University Affiliated Cancer Hospital Nanjing China; ^54^ Department of Clinical Pathology The First Affiliated Hospital of Jinan University Guangzhou China; ^55^ Institute of Respiratory Diseases Xinqiao Hospital, Third Military Medical University Chongqing China; ^56^ Department of Oncology, Key Laboratory of Clinical Cancer Pharmacology and Toxicology Research of Zhejiang Province Affiliated Hangzhou Cancer Hospital, Cancer Center, Zhejiang University School of Medicine Hangzhou China; ^57^ Department of Medical Oncology Sun Yat‐sen University Cancer Center, State Key Laboratory of Oncology in South China, Collaborative Innovation Center for Cancer Medicine Guangzhou China

**Keywords:** checkpoint inhibitor pneumonitis, Chinese experts consensus, immune checkpoint inhibitor‐related adverse effects

## Abstract

Immune checkpoint inhibitors (ICIs) have successfully treated a number of different types of cancer, which is of great significance for cancer treatment. With the widespread use of ICIs in clinical practice, the increasing checkpoint inhibitor pneumonia (CIP) will be a challenge to clinicians. To guide the diagnosis and treatment of CIP, we conducted in‐depth discussions based on the latest evidence, forming a consensus among Chinese experts on the multidisciplinary management of CIP.

## Graphical abstract

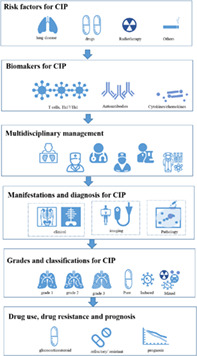

## INTRODUCTION

One of the complications of immune checkpoint inhibitor (ICI) therapy is checkpoint inhibitor pneumonitis (CIP). CIP can be manifested by focal or diffuse inflammation of the lung parenchyma accompanied by cough, shortness of breath, and hypoxemia or asymptomatic.^1^, ^2^ Sever CIP may cause fatalities.^3^ In clinical practice, the diagnosis and management of CIP is a great challenge.

In clinical trials, the incidence of CIP was reported to be 3% to 5%.^4^, ^5^, ^6^, ^7^, ^8^ When real‐world data were included, the incidence is up to 13% to 19%.^3^, ^9^, ^10^ The CPI incidence of non–small cell lung cancer (NSCLC) and renal cell cancer is higher than that of melanoma, which may be because of different tumor locations. A meta‐analysis showed that programmed cell death protein‐1(PD‐1) inhibitors had a higher risk of CIP than programmed death ligand‐1 (PD‐L1) inhibitors.^11^ In small cell lung cancer (SCLC) patients, the incidence of immune‐related adverse events (irAEs) such as pneumonitis caused by PD‐L1 inhibitors is lower than that of PD‐1 (4.3% vs. 2.1%).^12^

Previous study showed that the incidence of CIP in Japanese patients (8%–14%) is higher than that in non‐Asian population.^13^ To date, the different incidence of CIP among Asian and non‐Asian patient remains unclear. In patients treated with immunosuppressants, the level of early irAE is related to the clinical prognosis of patients.^14^ The overall mortality rate of adverse effects (AEs) caused by PD‐1/PD‐L1 inhibitors was 0.45%, and CIP was the most common cause (28.0%).^15^ A phase 3 Chinese patients trials showed that the incidence of CIP in the camerlizumab plus carboplatin group was similar to other combination therapies of ICIs and chemotherapy.^16^ Furthermore, all grade pneumonitis accounts for about 3%, and pneumonitis with grade more than 3 was ~2%.^16^ In another Chinese patient study, the incidence of CIP was 3.4% in the sintilimab plus pemetrexed group, and pneumonitis with grade more than 3 was 0.8%.^17^ In conclusion, although the overall incidence of CIP is not high, serious CIP needs to be taken seriously by clinicians.

## CONSENSUS 1: THE RISK FACTORS FOR CIP

A case–control study revealed the main risk factors of CIP through prior lung disease, thoracic radiotherapy (RT), and combination treatment of ICIs or epidermal growth factor receptor (EGFR) tyrosine kinase inhibitors (TKIs), with odds ratios (OR): 2.86, 3.34, and 2.73, respectively.^18^

### Previous lung disease

CIP is involved in several lung diseases, including chronic obstructive pulmonary disease (COPD), poor lung function, asthma, interstitial lung disease (ILD), pulmonary fibrosis, pneumothorax, and pleural effusion.^18^ It was reported that the frequency of CIP in patients with a history of asthma/COPD is higher than that in patients without a history of asthma/COPD (5.4% vs. 3.1%).^18^, ^19^ However, the number of CD4+ cells expressing PD‐1 is high in COPD patients. This may lead to a higher incidence of CIP and a longer progression‐free survival (PFS) in patients with mild COPD treated with ICIs.^20^ In addition, asthma may be associated with a higher grade of CIP, and smoking may augment this association.^21^ In addition to lower pretreatment percentage predicted forced expiratory volume in 1 s (FEV1pp), diffusion capacity of lung for carbon monoxide (DLCO) decline may be an early indicator of CIP.^22^ Therefore, routine pulmonary function testing with DLCO measurement during treatment may help risk stratify for CIP.^23^, ^24^ Isono et al.^25^ recently reported that idiopathic interstitial pneumonias (IIP) were a risk factor of CIP. Another study showed a significant difference in morbidity between patients with preexisting ILD and those without preexisting ILD (31% vs. 12%; *p* = 0.014).^26^ According to a retrospective analysis, the risk of CIP was closely associated with preexisting pulmonary fibrosis.^27^ Although prior lung diseases are not contraindications to PD‐1/PD‐L1, clinicians should be cautious when using ICIs for patients with previous lung disease.

### Combination with ICIs or EGFR‐TKIs

Chemotherapeutic drugs, TKIs, and other ICIs are often used in combination with ICIs. The incidence of CIP in patients with combined ICIs is higher than that in patients with ICI alone.^8^ For example, the combination of PD‐1 inhibitor and cytotoxic T lymphocyte antigen‐4 (CTLA‐4) inhibitor leaded to a significant increase in grade 1 to 5 pneumonitis compared with ICI alone^28^; the pneumonitis incidence rate in patients with EGFR‐TKI monotherapy was 4.59%, whereas it increased to 25.7% in patients received EGFR‐TKI combined with ICI treatment.^29^ Therefore, combination therapy may be a high‐risk factor that needs further safety assessment.

### Thoracic radiotherapy

Previous RT appears to be a potential risk factor for CIP. Among lung cancer patients who had pneumonitis, Aiad et al.^30^ reported that patients receiving immunotherapy combined with radiation were more serious than those receiving radiation therapy alone (55.7% vs. 36.2%). On the contrary, the PACIFIC study, which evaluated the effectiveness of durvalumab as maintenance therapy after definitive chemotherapy concurrent with RT for unresectable stage III NSCLC, demonstrated a moderate increase in pulmonary toxicity (durvalumab group vs. placebo group: 13.1% vs. 7.7%), and most of them were mild (grade 3–4 pneumonitis, durvalumab group vs. placebo group: 4.4% vs. 3.8%).^31^

### Other risk factors

The occurrence of CIP may be also related to age, smoking history, treatment history, Eastern Cooperative Oncology Group performance status (ECOG PS), virus infection, and histological type. The proportion of patients over 70 years old in CIP group was significantly higher than that in non‐CIP group (54.5% vs. 30.3%; *p* = 0.025). Compared with non‐smokers, former/current smokers had a higher incidence of pneumonitis.^32^ Pneumonitis of any grade may be more common in treatment‐naive patients.^33^ The incidence of CIP in patients with squamous histology is higher than that in patients with adenocarcinoma, and cytomegalovirus (CMV) infection may be an important trigger for CIP. It is important for patients with severe CIP to be vigilant against CMV infection.^34^ In patients with NSCLC, especially within 3 months of PD‐1 treatment, tumor invasion in the central airway was consistently associated with early‐onset CIP^34^, ^35^ In addition, ECOG PS ≥2 was closely correlated to the occurrence of CIP.^36^

## CONSENSUS 2: BIOMARKER OF CIP

Research is ongoing to determine which markers can be used to predict or diagnose CIP. Based on the mechanism of the disease, potential biomarkers mainly focus on cellular biomarkers, autoantibodies, cytokines/chemokines, and imaging biomarkers.

### Cellular biomarkers

Independent risk factors of grade 3/4 and lung irAEs includeed the elevated leukocyte count (*p* = 0.014, OR = 6.04) and decreased relative lymphocyte count (RLC) (*p* = 0.012, OR = 5.01).^37^ The incidence of CIP was high in patients with high peripheral blood eosinophil count before ICIs treatment.^38^

In bronchoalveolar lavage (BAL) fluid of patients with pulmonary symptoms after ICIs treatment, the number of T cells, especially interferon (IFN)ɤ + interleukin‐17 (IL‐17) − CD8+ T cells and CX chemokine receptor (CR) 3+ C‐C motif chemokine receptor (CCR) 6+ T helper (Th)17/Th1 cells increased. It is possible that CD8+ T cells and Th17/Th1 cells play an important role in CIP.^39^

### Autoantibodies

A panel of 5‐tumor associated autoantibodies (TAAbs) in plasma was selected to predict CIP. The 5‐TAAbs panel included p53, BRCA2, HUD, TRIM21, and NY‐ESO‐1. In contrast to negative patients, the incidence of CIP was significantly higher in patients with 5‐TAAbs positive than that in patients with 5‐TAAbs negative.^40^ Moreover, anti‐CD74 autoantibody was reported to correlate with the development of CIP, and may be used for early detected of CIP.^41^

### Cytokines/chemokines

Cytokines and chemokines can regulate the immune system. In CIP patients, elevated levels of IL‐6 and C‐reactive protein (CRP) in peripheral blood were considered potential biomarkers.^42^, ^43^, ^44^ The inflammation effect is influenced by these two factors. IFN‐ɤ may help to detect CIP after ICI‐based treatment.^45^ Moreover, increased levels of IL‐17A, IL‐35, IL‐6, IL‐10, neutrophil to lymphocyte ratio (NLR), platelet to lymphocyte ratio (PLR), and lactic dehydrogenase (LDH) levels or reduced absolute lymphocyte count (ALC) and albumin (ALB) levels were associated with the development of CIP.^46^, ^47^

Currently, although some progress has been made in the development of CIP markers, existing CIP studies are mainly retrospective and lacks accurate and effective biomarkers. It still needs sufficient prospective evidence to develop more meaningful biomarkers. In the future, it is necessary to carry out more research, especially prospective research, to improve our understanding of the underlying biology of CIP, to guide more precise and effective treatment strategies.

## CONSENSUS 3: THE MULTIDISCIPLINARY MANAGEMENT OF CIP

To improve the diagnosis and therapy of CIP, a multidisciplinary team consisting of pulmonologists, medical oncologists, thoracic radiologists, pathologists, thoracic surgeons, intensive care unit doctors, infectionist, immunologists, and rheumatologists with expertise in lung cancer, drug‐related pneumonitis, infectious diseases, and immunology was established to identify and prioritize knowledge gaps, and guide basic, translational, and clinical research focused on the etiology, diagnosis, and management of CIP.^48^, ^49^ Multidisciplinary will participate in translational and clinical trials, especially to provide clinical research samples such as serum, BAL, and lung pathologic specimens from affected patients, which may aid in identifying the biological differences that lead to variable clinical presentations, and may in turn, guide the development of phenotype‐specific targeted therapeutics to prevent or treat CIP. To avoid diagnostic delays, it is important to interact between disciplines. The complexity of CIP makes multidisciplinary collaborations essential for improving research and diagnosis.

## CONSENSUS 4: THE MANIFESTATIONS OF CIP

### Clinical manifestations

CIP has no typical and specific clinical features. Patients with CIP may be asymptomatic or accompanied by dyspnea and cough, whereas fever and chest pain are less common.^4^ The symptoms may be similar to respiratory tract infection, congestive heart failure, lymphangitis carcinomatosa, or another AE associated with systemic anti‐cancer therapy. Although fever and chest pain also sometimes occur, infectious pneumonia can also cause fever and chest pain. Therefore, the possibility of patients with fever should be excluded. In terms of disease course, CIP can manifest as acute, subacute, and chronic.^50^ Chronic CIP is defined as persistent or worsening pneumonia after steroid reduction, and it is necessary to carry out immunosuppression for more than 12 weeks after ICI discontinuation.^51^ Patients with early‐onset of CIP, within 6 weeks of ICI treatment, often have severe symptoms and poor prognosis, whereas patients with late‐onset of CIP, after 6 weeks of ICI treatment, often have few symptoms and better prognosis.^52^

The duration of CIP is variable from the beginning of ICI administration to withdrawal of the drug. Therefore, it is important to monitor CIP throughout the whole clinical process of ICI treatment.

### Imaging manifestations

Radiographic imaging is the most commonly used method for the diagnosis of pneumonitis. In 40% of patients, the pattern distribution was mixed and multifocal, and 75% had involvement of all lung lobes.^53^ According radiographic characteristics, CIP can be divided into organizing pneumonitis (OP), ground glass opacification/opacity (GGO), interstitial pneumonitis (IP), hypersensitive pneumonitis (HP), and other types.^53^, ^54^, ^55^ Nishino et al.^53^ screened 20 advanced cancer patients developed CIP from 10 different nivolumab trials, and identified 13 (65%) OP, three (15%) nonspecific interstitial pneumonitis (NSIP), two (10%) HP, and two (10%) acute interstitial pneumonia (AIP)/acute respiratory distress syndrome (ARDS). Another retrospective study reported a single‐characterized radiographic pattern, with 45% of patients having bilateral involvement and 86% having lung involvement away from the peri‐tumoral zone.^56^

In addition to radiographic imaging, bronchoscopy is also used for the diagnosis or auxiliary diagnosis of CIP, especially when X‐ray is difficult to diagnose. Bronchoscopy is helpful to diagnose CIP in patients with suspected infectious. Among the 12 patients diagnosed as CIP through bronchoscopy, 10 patients underwent BAL. Alveolitis was found in seven (87.5%) and OP was found in five (62.5%) patients. It also found acute lung injury and fibrosis.^57^ The largest series of bronchoalveolar lavage fluid (BALF) cell count results and 80% of BALF results showed a lymphocytosis of >15%. The proportion of lymphocytes in all 10 patients was higher than 20%.^57^ However, a subset of IIP, including idiopathic nonspecific interstitial pneumonitis (INP), cryptogenic organizing pneumonitis (COP) and connective tissue disorder‐related lung disease, is also predominant in lymphocytes and cannot be distinguished only by BALF findings.

Pathological examination may be helpful in understanding the specific mechanism of each classification if this clinical classification can be confirmed in a wider population.

### Pathological manifestations

The pathological manifestations of CIP are greatly concerned all along, but little is known. Most CIP specimens come from transbronchial lung biopsy (TBLB), and therefore, the sample is often small. There is also an inevitable heterogeneity in the pathological manifestations. In pathological results of nine CIP patients, the symptoms of all patients were nonspecific, and even two patients were asymptomatic. All cases showed bilateral ground glass or nodular opacities, and often accompanied by pleural effusion. Among them, seven patients were OP with subclinical or mild disease, and three patients admixed with vague non‐necrotizing airspace granulomas. Six patients with follow‐up were stable. Two patients died during treatment and follow‐up: one died from acute fibrinous pneumonitis, and the other died from diffuse alveolar damage. There was foamy macrophage vacuolization in all nine cases, and eosinophils were found in six cases. CIP presents with bilateral ground glass opacities or nodules and usually manifests with organizing pneumonitis histopathologically, often accompanied by vague non‐necrotizing airspace granulomas. Foamy macrophages and pneumocyte vacuolization are characteristic and rare eosinophils are often seen. In rare cases, acute fibrinous pneumonitis or diffuse alveolar damage may occur, which is fatal.^58^

## CONSENSUS 5: THE DIFFERENTIAL DIAGNOSIS OF CIP

As mentioned above, relatively nonspecific symptoms are insufficiency in vast majority of CIP patients. These symptoms include dyspnoea, chest discomfort, cough, and less commonly fever, which are common symptoms of other lung diseases. Hypoxia may lead to rapid progress of CIP. About one‐third of patients may be asymptomatic at onset. Because of the lack of specific clinical or radiologic markers, CIP is difficult to diagnose. Generally, CIP needs to be confirmed by exclusion diagnosis, but does not include infection, tumor progression, and radiation‐related pneumonitis. After ICI treatment, CIP should be considered for any new emergence of respiratory symptoms, especially dry cough, dyspnea, or decreased oxygen saturation.^59^

The diagnostic workup to identify an etiology should include detection of a source of infections such as nasal swab, sputum/urine culture, blood culture, detection of special pathogens such as fungus and uberculosis spot, chest radiography, bronchoscopy examination, and BAL examination.^4^, ^60^ Lung biopsy is not mandatory, and drugs and infection history occasionally help interpret the results. Use of diagnostic tests is related to the suspected pneumonitis grade.^61^

Common differential diagnoses of CIP include pulmonary infections, pulmonary embolism, diffuse alveolar damage (DAD), lung cancer with underlying progression, cancerous lymphangitis, pulmonary interstitial edema caused by heart failure, fulminant myocarditis, and radiation‐induced pneumonitis.^59^ During treatment with glucocorticosteroid (GCS) or other immunosuppressors, attention should be paid to secondary opportunistic infections arising from immune suppression. Opportunistic pulmonary infections, including tuberculosis (TB) pneumonia, aspergillosis, CMV pneumonia (CMVP), and pneumocystis jirovecii pneumonia (PJP), have been the foremost differential diagnoses of CIP in the NSCLC population. Notably, ICIs could cause special pathogen infections in some patients through inducing CIP. Aggressive lung biopsy was recommended to diagnose CIP in patients with NSCLC that mimicked the OP pattern or existed with the tumor invasion. The ICIs may cause inflammatory reaction in patients who previously irradiated fields with infiltrating lymphocytes and potential involvement related cytokines. Radiation recall pneumonitis (RRP) is characterized by inflammatory reaction within the previously treated radiation field after administration of specific treatment.^62^ Radiation pneumonitis occurs in the radioactive field, whereas CIP tends to occur nonsegmentally in both lung fields, especially outside the regions of high‐dose chest radiation. In the majority of RRP cases, the area of pneumonitis matches the irradiated area. Xuguang Chen et al.^63^ found that patients with bilateral computed tomography (CT) changes involving at least three lobes were more likely to develop CIP, whereas patients with unilateral CT changes with clear boundaries were more likely to develop radiation pneumonitis. After RT or ICI, severe pneumonitis is associated with bilateral and multifocal CT changes.^63^ Quantitative CT radiomics and machine learning may help determine the cause of pneumonia for patients and improve personalized clinical treatment.^64^

## CONSENSUS 6: THE CIP GRADING AND CLINICAL CLASSIFICATION

Imaging manifestations and clinical symptoms are usually used for CIP grating. According to the National Comprehensive Cancer Network guidelines,^65^ CIP is graded by the combination of clinical manifestations and radiological findings as follows (Table 1).

**TABLE 1:** The grades of CIP based on clinical manifestations and radiological findings

Grades	Clinical manifestations	Radiological findings
Grade 1	Asymptomatic	One lobe of the lung or <25% of the lung parenchyma
Grade 2	New respiratory symptoms, or aggravation of existing symptoms such as shortness of breath, cough, chest pain, fever, and increased oxygen requirements	Lesions affect 25%–50% of the lung parenchyma
Grade 3	Severe symptoms, limited daily activities	Lesions affect all lung lobes or >50% of the lung parenchyma
Grade 4	Life‐threatening respiratory damage	

Abbreviation: CIP, checkpoint inhibitor pneumonia.

Grade 1 is asymptomatic. The lesion is confined to one lobe of the lung or <25% of the lung parenchyma. Grade 2 includes new respiratory symptoms or aggravation of existing symptoms, including shortness of breath, cough, chest pain, fever, and increased oxygen requirements. On the chest CT, lesions affect 25% to 50% of the lung parenchyma. Grade 3 includes severe symptoms and limited daily activities. Lesions affect all lung lobes or >50% of the lung parenchyma. Grade 4 is life‐threatening respiratory damage.

However, the course and pathology type of CIP were not considered in the guidelines. Even if the patient is grade 2 to 3 at the time of diagnosis, and even for patients with rapid progression or severe imaging manifestations such as diffuse alveolar damage, close monitoring is recommended. In addition, CIP is not well classified according to clinical factors. The clinical classification of CIP may help to formulate treatment strategies and predict the tumor response to ICIs.^66^ Based on clinical factors, CIP can be classified into three clinical subtypes, as shown below (Table 2):

1. Pure type (PT): defined as idiopathic, with or without autoimmune disease. In PT group, most patients were grade 1 to 2, with imaging COP, GGO. The treatment of CIP in the PT group basically followed the guidelines based on systemic glucocorticoids, supplemented by other immunosuppressive agents when necessary.
2. Induced type (IT): CMV or Epstein–Barr virus (EBV) is reactivated, RT is reactivated, and there is no evidence of organ damage caused by virus or RT. In IT group, most patients were grade 3 to 4, with imaging GGO (AIP). The IT participants received non‐thoracic RT and subsequently developed CIP. In addition, pneumonitis did not occur in these patients during multiple courses of immunotherapy, but developed pneumonia after RT. GGO has the highest proportion in the IT. The incidence of AIP‐ARDS in the IT group was higher than that in the other two groups. For IT patients, in addition to corticosteroids and supportive treatment, antiviral therapy for virus‐induced CIP and anti‐fibrotic therapy for RT‐ induced CIP can be considered.
3. Mixed type (MT): combined with infectious pneumonia (such as bacteria, fungus, or other organisms), tumor progression, or radiation‐related pneumonitis. In MT group, CIP of most patients were grade 2 to 4, with imaging NSIP, GGO. The MT was characterized by a combination of symptoms and imaging changes associated with infection, tumor progression, or radiation pneumonitis. Patients in MT group developed CIP with CMVP, which was diagnosed by positive CMV culture in BALF or tissue, CMV‐DNA in BALF, or CMV inclusion bodies in lung tissues. For MT patients, antibiotic treatment for co‐infection, antitumor treatment for co‐tumor progression, and anti‐fibrotic therapy for patients complicated with co‐radiation pneumonitis can be considered.

**TABLE 2:** The clinical subtypes of CIP based on clinical factors

Subtypes	Clinical factors	Grades	Imaging features	Treatments
Pure type	Idiopathic, with or without autoimmune disease	Grade 1–2	COP, GGO	Basically followed the guidelines basing on systemic glucocorticoids, supplemented by other immunosuppressive agents when necessary
Induced type	CMV or EBV reactivation, radiotherapy reactivation, without evidence of organ damage caused by virus or radiotherapy	Grade 3–4	GGO, AIP	Corticosteroids and supportive treatment, antiviral therapy (for virus‐induced CIP), and anti‐fibrotic therapy (for radiotherapy‐ induced CIP) can be considered
Mixed type	Combined with infectious pneumonia (bacteria, fungus, or other organisms), tumor progression, or radiation‐related pneumonitis	Grade 2–4	NSIP, GGO	Antibiotic treatment (for co‐infection), antitumor treatment (for co‐tumor progression), and anti‐fibrotic therapy (for patients complicated with co‐radiation pneumonitis) can be considered

Abbreviations: AIP, acute interstitial pneumonia; CIP, checkpoint inhibitor pneumonia; COP, cryptogenic organizing pneumonitis; CMV, cytomegalovirus; EBV, Epstein–Barr virus; GGO, ground glass opacification/opacity; NSIP, nonspecific interstitial pneumonitis.

## CONSENSUS 7: THE USE OF GLUCOCORTICOSTEROID

GCS is the main treatment for CIP. Most studies have found that corticosteroid therapy can improve or resolve symptoms in patients with CIP, especially those with lower‐grade disease.^67^ In 70% to 80% CIP patients, regular GCS treatment can control the disease.^68^ For patients with grade 1 CIP, close monitoring is recommended, whereas GCS treatment should be considered if clinical progression is observed. Prednisolone is the most commonly used GCS in treatment of CIP. The equivalent dose of prednisolone (1–2 mg/kg/day) is recommended for grade 2 to 3 CIP, whereas intravenous GCS is recommended for more severe or acute CIP. It is recommended to gradually reduce GCS after clinical symptom remission are relieved. GCS treatment usually lasts for 6 to 8 weeks, but does not exceed 12 weeks. For CIP, clinical improvements should be assessed within 48 to 72 hours of GCS treatment. Patients receiving GCS treatment should be considered infectious diseases. In addition, blood pressure, glucose, and electrolytes should be monitored. The total duration of GCS treatment in most CIP patients is ~8 weeks, and the duration of the initial steroid dose is usually <3 weeks. Therefore, treatment of pneumocystis carinii is generally not required, except for patients who take 20 mg GCS per day for >6 weeks. It is possible to supplement calcium and vitamin D3 regularly.

## CONSENSUS 8: STEROID‐REFRACTORY/RESISTANT CIP

Some patients are refractory or become resistant to steroids. According to the indications of additional immunomodulators, patients were divided into two subgroups: (i) steroid refractory, which are patients whose pneumonia did not improve or worsen after systemic steroid treatment; (ii) steroid resistant, which are patients who initially responded to steroids, but subsequently developed recurrent pneumonitis when steroid gradually decreased in the absence of immune checkpoint re‐challenge. Patients with steroid‐refractory pneumonitis had more severe disease than those with steroid‐resistant pneumonitis (grade 3–4, 100% vs. 29%, *p* = 0.0002). Compared with steroid‐resistant patients, steroids‐refractory patients exhibited earlier onset and fewer durable responses.^69^, ^70^ Steroid‐refractory CIP accounts for 18.5% of patients with multidisciplinary care.^71^ The guidelines provide a list of additional immune modulators, however, evidences to guide expectations for patients and providers is limited. Infliximab, mycophenolate mofetil, immunoglobulin, and cyclophosphamide are some additional immune modulating options recommended for patients whose steroids are ineffective. Currently, these agents are mainly used based on experience of treating other irAEs and other inflammatory lung diseases. According to prospective comparative data, it is unclear what the exact role of below listed agents are.^70^, ^72^

1. Intravenous immunoglobulin (IVIG). IVIG can be used to neutralize antigens with good safety. It is especially preferred in patients with potential infection. The dosage of IVIG is 20 g per day for 3 consecutive days or 10 g per day for 5 consecutive days and can be used repeatedly if necessary. The mortality of patients treated with IVIG is low, and the oxygenation of patient can be improved.^71^
2. Antitumor necrosis factor (TNF‐α). Infliximab, a TNF‐α inhibitor, has been used successfully to treat steroid‐ refractory ICI‐colitis, and the effect of infliximab‐ containing regimen treatment is poor.^71^ Prospective data should be obtained from clinical trials in this area to identify the optimum immunosuppressive approach for steroid‐ refractory CIP. A multicenter‐randomized study of infliximab versus IVIG in steroid‐refractory CIP treatment is ongoing (NCT04438382).
3. IL‐6 receptor inhibitor. Tocilizumab is a humanized monoclonal antibody against IL‐6 receptors, which can block inflammatory cascade reaction and reduce the systemic inflammatory reaction and lung damage. Tocilizumab may be a therapeutic option, however, randomized trials are still needed to better elucidate the relative efficacy and safety.^73^
4. Antifibrotic drugs. Antifibrotic drugs may be effective in patients with CIP. Nintedanib is a tyrosine kinase inhibitor, which can efficiently slow down the progression of idiopathic pulmonary fibrosis (IPF) and has an acceptable tolerability profile.^74^ The pembrolizumab‐related pneumonitis was significantly improved after adding nintedanib in an advanced NSCLC patient. The patient did not obtain any clinical symptom relief with methylprednisolone alone. Pirfenidone is also an effective drug for the treatment of pulmonary fibrosis (PF). A clinical trial of pirfenidone combined with methylprednisolone versus methylprednisolone in the treatment of CIP is recruiting (NCT05280873).
5. Other immunosuppressors. Immunosuppressors such as mycophenolate mofetil and cyclophosphamide are also recommended in some practice guidelines. However, there is still a lack of study on patients treated with immunosuppressors, and this limits our ability to identify truly desirable immunosuppressive therapies.

We recommend corticosteroids as the first line treatment for most CIP. It is recommended to obtain additional details of the immunohistopathology whenever possible. Biopsy should at least be performed on the clinically affected end organ, which will provide information for subsequent targeted therapies. The immunopathogenic mechanisms should be studied in more detail as far as possible, including detailed immunohistochemistry of the affected end organ, peripheral blood flow cytometry, measurement of levels of autoantibodies and assessment of peripheral blood cytokines (such as IL‐1, IL‐6, TNF, and IL‐17). These data may enable us to make more accurate therapeutic decisions when selecting the optimal targeted therapies.^75^ To develop biologically‐informed treatment for steroid‐resistant/resistant CIP, it is necessary to better understand the pathophysiology of CIP and recommend relevant clinical and basic trials.

## CONSENSUS 9: THE PROGNOSIS OF CIP

In CIP patients, Suresh et al.^3^ demonstrated that although ICIs did not significantly affect short‐term survival including disease control rate, overall response rate, and PFS, it would decrease the overall survival(OS) by 10 months. Another study reported that CIP increased PFS and OS, and 25% of patients continued to have no tumor growth after treatment discontinuation.^76^ In real world studies, CIP is associated with poor prognosis.^3^, ^9^ On the other hand, the efficacy of ICI in patients with irAEs has improved.^77^ The severe grade of CIP (≥grade 3) is associated with the reduction of PFS and OS.^10^

## AUTHOR CONTRIBUTIONS

Lu Si, Wenfeng Fang and Yong Song participated in the design of the expert consensus. Wenxian Wang, Qian Wang and Chunwei Xu conceived of the expert consensus, and participated in its design and other authors coordination and helped to draft the expert consensus. All authors read and approved the final manuscript.

## CONFLICTS OF INTEREST

The authors declare that they have no potential conflicts of interest, financial interests, relationships and affiliations relevant to the subject of their manuscript.
